# Spread of Oropouche Virus into the Central Nervous System in Mouse

**DOI:** 10.3390/v6103827

**Published:** 2014-10-10

**Authors:** Rodrigo I. Santos, Lézio S. Bueno-Júnior, Rafael N. Ruggiero, Mariana F. Almeida, Maria L. Silva, Flávia E. Paula, Vani M. A. Correa, Eurico Arruda

**Affiliations:** 1 Department of Cell Biology, University of Sao Paulo, School of Medicine at Ribeirao Preto, Ribeirao Preto, 14049-900, Brazil; E-Mails: rodrigoivo97@gmail.com (R.I.S.); ma_fefa@hotmail.com (M.F.A.); mlsilva@usp.br (M.L.S.); escremim@gmail.com (F.E.P.); vanimariaac@yahoo.com.br (V.M.A.C.); 2 Department of Pathology, University of Texas Medical Branch at Galveston, Galveston, TX 77555, USA; 3 Department of Neurology and Behavioral Sciences, University of Sao Paulo, School of Medicine at Ribeirao Preto, Ribeirao Preto, 14049-900, Brazil; E-Mails: lezioneuro@gmail.com (L.S.B.-J.); rafaruggiero@yahoo.com.br (R.N.R.)

**Keywords:** Oropouche virus, neuropathogenesis, brainstem

## Abstract

Oropouche virus (OROV) is an important cause of arboviral illness in Brazil and other Latin American countries, with most cases clinically manifested as acute febrile illness referred to as Oropouche fever, including myalgia, headache, arthralgia and malaise. However, OROV can also affect the central nervous system (CNS) with clinical neurological implications. Little is known regarding OROV pathogenesis, especially how OROV gains access to the CNS. In the present study, neonatal BALB/c mice were inoculated with OROV by the subcutaneous route and the progression of OROV spread into the CNS was evaluated. Immunohistochemistry revealed that OROV infection advances from posterior parts of the brain, including the periaqueductal gray, toward the forebrain. In the early phases of the infection OROV gains access to neural routes, reaching the spinal cord and ascending to the brain through brainstem regions, with little inflammation. Later, as infection progresses, OROV crosses the blood-brain barrier, resulting in more intense spread into the brain parenchyma, with more severe manifestations of encephalitis.

## 1. Introduction

Oropouche virus (OROV), of the family *Bunyaviridae,* genus *Orthobunyavirus*, serogroup Simbu, is an important causative agent of arboviral febrile illness in Brazil. Over half a million cases of Oropouche fever have occurred in Brazil since the 1960s. There have been reports of OROV outbreaks also in other countries of South America and the Caribbean, such as Panama, Peru, Suriname and Trinidad and Tobago [1]. The vast majority of cases of OROV infection are clinically manifested as Oropouche fever, a dengue-like illness with fever, myalgia, headache, arthralgia, skin rash and malaise, associated with viremia in the initial 5–6 days. Recovery usually takes 2–3 weeks without known sequelae or mortality, but symptoms can occasionally last longer, and relapses are common [2]. Despite its importance for public health, little is known about the pathogenesis of OROV infection.

OROV is detectable in the cerebrospinal fluid of patients with clinical meningitis. In a hospital-based study in Manaus, a large city in the Amazon, 3 out 49 (6.1%) patients with viral meningitis had OROV detected in the CSF [3]. Neurological symptoms have been reported sporadically in patients with OROV fever, suggesting that OROV can cause neuropathogenesis [2,4,5,6]. During an outbreak situation in northern of Brazil, 22 cases of meningitis were reported in 292 patients (7.5%) with OROV fever [6]. OROV consistently infects experimental animals, especially mice and hamsters, by intracerebral inoculation [7,8,9]. However, CNS infection upon intracerebral inoculation bypasses all barriers present in naturally infected hosts. In order to evaluate CNS involvement during experimental infections that most closely resemble natural routes of infection, we have developed experimental models inoculated by the subcutaneous route, both in neonatal mice and hamsters [10,11]. Those studies revealed that the OROV experimental inoculation of hamsters induces systemic infection with development of paralysis and death in roughly one third of the animals, along with conspicuous involvement of the liver and brain as target organs [11]. However, because hamsters are non-isogenic animals, they are unsuitable for detailed immunological assessment of pathogenesis using commercially available reagents. Therefore, alternatively, isogenic BALB/c mice were tried as more appropriate experimental hosts of OROV infection.

In a previous study, neonate BALB/c mice were subcutaneously inoculated with OROV [10]. The vast majority of infected animals on the 5th day post inoculation developed disease characterized mainly by lethargy and paralysis, with progression to death within 10 days. Viral replication was documented in brain neurons by *in situ* hybridization, immunohistochemistry and virus titration. Despite the severe CNS disease, histopathology was mild in the brain and spinal cord, with little inflammation. There was obvious glial reaction and astrocyte activation in spinal cord and brain, where OROV induced apoptosis of neurons and microglial activation, accompanied by mild meningitis. Spleen hyperplasia was also found [10]. Of note, apoptosis induced by OROV has been observed both *in vivo* and *in vitro* [10,12].

In the present study, a more detailed investigation was done on the progression of OROV spread throughout the nervous system.

## 2. Materials and Methods

The OROV strain BeAn 19991, originally a gift from Dr. Luis Tadeu Figueiredo (University of Sao Paulo School of Medicine, Ribeirao Preto, SP, Brazil), was propagated by one passage in Swiss suckling mouse brain, followed by 3 passages in HeLa cells. The resulting stock was titrated by cytopathic effect induction in Vero cells and expressed as 50% infectious dose (TCID_50_). Twenty one day-old mice were inoculated subcutaneously with 10^6.25^ TCID_50_ of OROV, either on the dorso-lumbar area or on the ventral thorax above the rib cage. After euthanasia by ketamine (200 mg/kg)/xylasine (150 mg/Kg) and cervical dislocation, animals were perfused with PBS and then with buffered 3.7% formaldehyde pH 7.4. Brain, spinal cord and ribs were dissected for fixation and paraffin embedding, using established protocols [13]. Two sets of animals were used: In the first group 10 animals were inoculated and euthanized if signs of severe sickness were observed, regardless of the time elapsed before disease onset. Signs of sickness included paralysis, inability to breastfeed and shaking chills. In the second group, eleven animals were euthanized 3 or 4 days post-infection (dpi) regardless of the presence of signs of disease, to evaluate progression of the involvement of the infection kinetics and the route of OROV infection. The study was approved by the University of São Paulo Committee on Care and Use of Laboratory Animals (protocol #165/2008).

In both groups, immunohistochemistry for OROV antigen was done in 6 µm coronal sections using anti-OROV rabbit serum [10]. Intervals of 30–45 µm were used, to cover the whole extension of the brain from the rostral end until the beginning of the spinal cord. For data analysis (Table 1 and Table 2), percentages of OROV positive cells per visual field (400×) were calculated. CNS regions were ranked according to the intensity of positivity for OROV antigen as: + for 1% to 25% of positive cells; ++ for 25% to 50% of positive cells; +++ for 50% to 75% of positive cells; and ++++ for over 75% of positive cells.

**Table 1:** Brain regions infected by OROV in animals free of signs and symptoms euthanized at 3, 4 or 6 dpi.

		ANIMAL	20D/2011	20C/2011	13E/2011	13*/2011	007/2011	006/2011	12A/2011	12B/2011	12C/2011	008/2011	21B/2011	21A/2011
			3dpi	3dpi	3dpi	3dpi	3dpi	3dpi	4dpi	4dpi	4dpi	4dpi	6dpi	6dpi
Telencephalon:	Neocortex	Auditory	0 (0/0)	0 (0/0)	0 (0/0)	0 (0/0)	0 (0/0)	++++ (++++/++++)	na	na	na	na	0 (0/0)	0 (0/0)
		Motor	++ (0/++)	+ (0/++)	0 (0/0)	0 (0/0)	0 (0/0)	+++ (+/++++)	0 (0/0)	0 (0/0)	0 (0/0)	+ (0/++)	0 (0/0)	0 (0/0)
		Prefrontal	0 (0/0)	0 (0/0)	0 (0/0)	0 (0/0)	0 (0/0)	0 (0/0)	0 (0/0)	0 (0/0)	0 (0/0)	++++ (+++/++++)	0 (0/0)	0 (0/0)
		Retrosplenial	+ (0/++)	++ (+/++)	0 (0/0)	0 (0/0)	0 (0/0)	++++ (++++/++++)	0 (0/0)	0 (0/0)	0 (0/0)	+++ (+/+++++)	0 (0/0)	0 (0/0)
		Somatosensory	+ (0/+)	+ (0/+)	0 (0/0)	0 (0/0)	0 (0/0)	++ (+/++)	0 (0/0)	0 (0/0)	0 (0/0)	++++ (+/++++)	0 (0/0)	0 (0/0)
		Visual	na	+ (+/+)	0 (0/0)	0 (0/0)	0 (0/0)	+ (+/+)	0 (0/0)	0 (0/0)	0 (0/0)	* (0/*)	0 (0/0)	0 (0/0)
		Piriform	* (0/*)	+ (0/+)	0 (0/0)	0 (0/0)	0 (0/0)	+++ (+/++++)	0 (0/0)	0 (0/0)	0 (0/0)	++++ (++++/++++)	0 (0/0)	0 (0/0)
	Hippoc	CA1	0 (0/0)	0 (0/0)	0 (0/0)	0 (0/0)	0 (0/0)	++++ (++++/++++)	0 (0/0)	0 (0/0)	0 (0/0)	+ (+/+)	0 (0/0)	0 (0/0)
		CA3	0 (0/0)	0 (0/0)	0 (0/0)	0 (0/0)	0 (0/0)	++++ (++++/++++)	0 (0/0)	0 (0/0)	0 (0/0)	++++ (++++/++++)	0 (0/0)	0 (0/0)
		Dentate gyrus	0 (0/0)	0 (0/0)	0 (0/0)	0 (0/0)	0 (0/0)	++++ (++++/++++)	0 (0/0)	0 (0/0)	0 (0/0)	+++ (+++/+++)	0 (0/0)	0 (0/0)
	Subcortical nuclei	Nucleus accumbens	0 (0/0)	0 (0/0)	na	0 (0/0)	0 (0/0)	++++ (++++/++++)	0 (0/0)	0 (0/0)	0 (0/0)	++++ (++++/++++)	0 (0/0)	0 (0/0)
		Amygdaloid complex	* (*/*)	* (*/*)	0 (0/0)	0 (0/0)	na	+ (+/++)	na	na	na	na	0 (0/0)	0 (0/0)
		Anterior olfactory nucleus	na	0 (0/0)	na	0 (0/0)	0 (0/0)	0 (0/0)	0 (0/0)	0 (0/0)	0 (0/0)	+++ (*/++++)	+ (0/+)	na
		Striatum	0 (0/0)	0 (0/0)	0 (0/0)	0 (0/0)	0 (0/0)	+++ (+++/+++)	0 (0/0)	0 (0/0)	0 (0/0)	++++ (++++/++++)	0 (0/0)	0 (0/0)
		Septal nuclei	0 (0/0)	0 (0/0)	0 (0/0)	0 (0/0)	0 (0/0)	++++ (+++/++++)	na	na	na	++++ (++++/++++)	0 (0/0)	0 (0/0)
		Ventral pallidum	na	na	0 (0/0)	na	na	na	na	na	na	na	na	na
Diencephalon		Dorsal thalamus	0 (0/0)	0 (0/0)	0 (0/0)	0 (0/0)	0 (0/0)	++ (+/++++)	0 (0/0)	0 (0/0)	0 (0/0)	++++ (++++/++++)	0 (0/0)	0 (0/0)
		Hypothalamus	0 (0/0)	+ (0/+)	0 (0/0)	0 (0/0)	0 (0/0)	+++ (++/++++)	na	na	na	++++ (++++/++++)	0 (0/0)	0 (0/0)
Mesencephalon		Inferior colliculus	0 (0/0)	+ (*/+)	0 (0/0)	0 (0/0)	0 (0/0)	+ (0/+)	0 (0/0)	0 (0/0)	0 (0/0)	+ (0/+)	0 (0/0)	0 (0/0)
		Periaqueductal grey	+ (0/+)	+ (*/+)	0 (0/0)	0 (0/0)	0 (0/0)	++++ (++/++++)	0 (0/0)	0 (0/0)	0 (0/0)	+++ (+/++++)	++(++/++)	0 (0/0)
		Superior colliculus	0 (0/0)	+ (+/+)	0 (0/0)	0 (0/0)	na	++++ (+++/++++)	0 (0/0)	0 (0/0)	0 (0/0)	na	0 (0/0)	0 (0/0)
		Other regions	0 (0/0)	+++(0/+++)	0 (0/0)	0 (0/0)	+ (0/+++)	++++ (++/++++)	0 (0/0)	0 (0/0)	0 (0/0)	++++ (++++/++++)	0 (0/0)	++ (0/++)
Rhombencephalon		Pons	0 (0/0)	++ (+/++)	na	0 (0/0)	0 (0/0)	++++ (+++/++++)	na	na	na	++ (+/+++)	0 (0/0)	0 (0/0)
		Cerebellum	na	+ (0/+)	0 (0/0)	0 (0/0)	0 (0/0)	+ (+/++)	0 (0/0)	0 (0/0)	0 (0/0)	0 (0/*)	0 (0/0)	0 (0/0)

**Table 2:** Brain regions infected by OROV in animals with apparent symptoms.

		ANIMAL	20F/2011	26F/2011	26C/2011	18A/2011	001/2010	002/2010	p1/2011	26B/2011	26A/2011
			7dpi	6dpi	6dpi	6dpi	6dpi	6dpi	6dpi	5dpi	4dpi
Telencephalon:	Neocortex	Auditory	+ (0/++)	na	+++ (+/++++)	+ (0/+++)	++ (++/++)	++ (++/++)	+ (+/+)	+ (*/+)	+ (0/+)
		Motor	+ (*/+)	++ (+/+++)	+++ (++/+++)	+ (0/+)	+++ (+/++++	+ (+/+)	++++(++++/++++)	++++(++++/++++)	+ (0/++)
		Prefrontal	+ (*/+)	+ (+/++)	+ (+/++)	0 (0/0)	++++(++++/++++)	+ (+/+)	+++ (++/+++)	na	+ (*/++)
		Retrosplenial	+ (+/+)	+++ (++/++++)	+ (0/++)	+ (0/+)	++ (++/+++)	++ (++/++)	++++(++++/++++)	++ (+/++++)	+ (+/++)
		Somatosensory	+ (0/+)	+ (+/+)	+++ (0/++++)	++ (0/+++)	++++ (++/++++)	* (*/*)	++++(++++/++++)	+++ (+/++++)	+ (0/++)
		Visual	+ (0/+)	+ (+/+)	+ (0/++)	++(+/++)	++ (++/++)	* (*/*)	na	+++ (+/++++)	+++(+++/+++)
		Piriform	+ (+/+)	+ (+/+)	+ (0/+)	+ (+/++)	++ (+/+++)	+ (+/++)	++ (++/++)	++ (++/++)	+ (+/+)
	Hippoc.	CA1	++ (*/++)	+ (0/+)	++ (+/+++)	++ (0/+++)	+++ (+++/+++)	+ (+/+)	++++(++++/++++)	0 (0/0)	+ (*/+)
		CA3	++ (+/++)	++ (+/++)	++ (+/+++)	+ (0/++)	+++ (+++/+++)	+ (+/+)	++ (++/++)	0 (*/0)	++ (*/+++)
		Dentate gyrus	+ (*/+)	+ (+/+)	+ (+/+)	+ (0/+)	+ (+/+)	+ (+/+)	+ (+/+)	+ (0/+)	+ (+/+)
	Subcortical nuclei	Nucleus accumbens	+ (0/++)	+ (+/+)	+ (+/+)	* (*/*)	0 (0/0)	+ (*/+)	+ (+/+)	+ (+/+)	0 (0/0)
		Amygdaloid complex	* (0/*)	na	+ (+/+)	na	na	+++(+++/++++)	+ (+/+)	0 (0/0)	0 (0/0)
		Anterior olfactory nucleus	na	+ (+/+)	na	0 (0/0)	na	na	na	na	+ (0/+)
		Striatum	+ (*/+)	+ (+/+)	+ (0/+)	+ (*/+)	+ (0/++)	+ (+/+++)	+ (+/++)	+ (0/+)	* (*/*)
		Septal nuclei	++ (+/++)	na	+ (+/+)	++ (++/+++)	+++ (+++/+++)	na	+ (+/+)	+++ (++/++++)	0 (0/0)
		Ventral pallidum	na	na	na	na	na	na	na	na	0 (0/0)
Diencephalon		Dorsal thalamus	+ (+/+)	na	+ (0/+)	+ (0/++)	+ (+/+)	+ (+/+++)	++ (+/++)	++ (*/++++)	* (0/*)
		Hypothalamus	+ (+/+)	+ (+/+)		+ (0/++)	0 (0/0)	+++ (+/++++)	++ (+/+++)	++ (0/++)	+ (0/+)
Mesencephalon		Inferior colliculus	0 (0/0)	+ (+/+)	+ (*/+)	0 (0/0)	na	na	na	0 (0/0)	* (*/*)
		Periaqueductal grey	++ (+/+++)	+++ (++/++++)	++ (+/++)	++ (++/++)	++ (+/++0	+ (+/++)	+ (0/+)	+ (*/++)	++ (*/+++)
		Superior colliculus	0 (*/0)	++++(++++/++++)	0 (0/0)	* (*/*)	+ (+/++)	+ (+/*)	0 (0/0)	+ (*/+)	+ (+/+)
		Other regions	++ (+/++)	++++ (+++/++++)	++ (++/++++)	+++ (*/+++)	++ (+/+++)	++ (+/+++)	* (0/*)	+++ (++/++++)	* (*/*)
Rhombencephalon		Pons	0 (*/0)	+++ (+/++++)	+ (+/+)	+++ (+++/+++)	na	na	na	++++(++++/++++)	+ (+/+)
		Cerebellum	* (*/0)	++ (+/++)	+ (*/+)	0 (0/0)	+ (+/+)	0 (0/0)	+ (+/+)	0 (0/0)	na

Results are shown as percentage groups: 0 no positive cells, * 1 to 5 positive cell in a field, + until 25% of positive cells; ++ 25% to 50% of positive cells; +++ 50% to 75% of positive cells; ++++ 75% to 100% of positive cells. The result is an average of all photomicrographs taken in one brain region. Complete absence of positive cells was denoted as (0), and the presence of only sporadic positive cells, less than 1%, was denoted with the sign *. The maximum and minimum positivity for each brain region are shown within parentheses (minimum/ maximum). ‘Hippoc.’ refers to hippocampus; ‘na’ means “not analyzed”.

We photographed one to four separate frames from each brain region depending on its size, and the percentage of positivity was counted in all frames. Brain regions examined were: (1) Telencephalon-neocortex (auditory, motor, piriform, prefrontal, retrosplenial, somatosensory, and visual cortices), hippocampus (CA1, CA3, and dentate gyrus), and subcortical nuclei (amygdaloid complex, anterior olfactory nucleus, nucleus accumbens, septal nuclei, striatum, and ventral pallidum); (2) Diencephalon (dorsal thalamus, and hypothalamus); (3) Mesencephalon (inferior colliculus, periaqueductal gray, and superior colliculus); and (4) Rhombencephalon (cerebellum, and pons). For visual referencing of brain areas, we used the Allen Institute’s open-access atlas of the mouse brain [14]. Representative histological images were organized according to regions/structures.

For blood-brain barrier permeability assay nine mice were euthanized as previously described and perfused by intra-cardiac injection of 1% Evan’s blue followed by PBS. The whole brains were removed and weighted and immersed overnight in formamide (1 mL/100 mg of brain). Evan’s blue solubilized from the brain by formamide was quantified in a spectrophotometer at the wavelength of 560 nm. Animals with OD values higher than controls were considered to have damaged blood-brain barrier.

## 3. Results and Discussion

In animals euthanized three to four days after infection, OROV antigen was found in 7 of 12 mice, and in all 7 animals OROV antigen was detected throughout the brainstem (here, brainstem refers to the mesencephalon, or midbrain, and the pons). In particular, the periaqueductal gray was found to be OROV-positive in 5 animals. This may suggest that the periaqueductal gray could be an important OROV gateway from the cerebral ventricular system into the brain. Both the inferior and superior colliculi, as well as the pons, were positive for OROV in 3 of 12 animals. Importantly, none of the animals positive for OROV in this group presented clinical manifestations. This lack of obvious neurological deficit in spite of heavy neuronal staining for viral antigen, suggests that OROV replication in neurons may occur with relatively little functional neuronal impairment. The OROV antigen positivity distribution and intensity per animal are summarized in Table 1.

**Figure 1:**
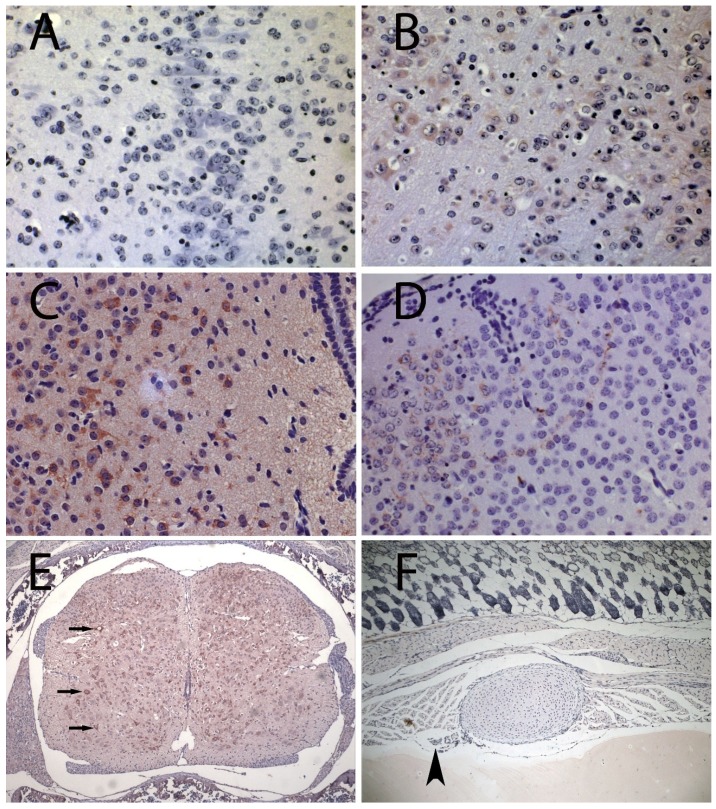
Immunohistochemistry for Oropouche virus (OROV) antigen in sections of brain, spinal cord and chest of BALB/c mice. (**A**) brainstem (bregma -7) from control (400×); (**B**) brainstem (bregma -9) from OROV-inoculated mouse at 3 dpi (400×); (**C**) Periaqueductal grey matter from OROV-infected mouse at 6 dpi (400×); (**D**) Visual cortex from OROV-infected mouse at 6 dpi (400×); (**E**) Spinal cord from OROV-infected mouse at 6 dpi showing infected motor neurons (arrows) (100×); (**F**) Chest of OROV‑infected mouse at 1 dpi showing intercostal nerve next to rib (arrowhead) without visible staining (400×). OROV positive cells are evidenced by the presence of tan-brown staining (B–E).

OROV antigen distribution over time suggests that, in this model, OROV infection ascends from the brainstem to the cerebral cortex. As expected, the brainstem was more heavily infected in its most posterior portions (Figure 1A,B). OROV antigen was present in the telencephalon in four of 12 animals, and 2 of them had brainwide OROV infection. The only exceptions were the two more rostral analyzed regions: the prefrontal cortex and olfactory tubercle, both without any discernible viral antigen. Because these regions are the farthest from the brainstem, probably OROV could not reach them prior to the euthanasia. The other two animals had detectable OROV antigen in different regions of the telencephalon, albeit in lower percentages of infected cells.

With regard to the animals that showed signs of sickness (paralysis, inability to breastfeed and shaking chills), OROV antigen was detected throughout the entire brain (Table 2). The infection was disseminated in the brainstem, diencephalon and telencephalon. In the telencephalon, the infection reached the prefrontal cortex and olfactory tubercle. In the cerebellum, the virus was found in five of nine animals, yet at lower percentages of infected cells when compared with the brainstem and telencephalon. Interestingly, in animals that developed disease, the distribution of OROV antigen did not suggest a pattern of ascending spread, as observed in animals euthanized prior to disease onset. We hypothesize that in animals that became sick (most with disease onset later than 4 days) OROV may have diffusely reached all the different brain areas by a non-neural route, perhaps owing to blood brain barrier breach. Indeed, OROV antigen positivity in areas near the meninges and in the periaqueductal grey matter corroborates a presumptive hematogenous access of OROV into the brain at later times post infection (Figure 1C,D). In fact, sick animals had greater Evans blue staining in the brain as compared to animals without disease manifestations, indicating that the blood-brain barrier leakage can be related to the severity of the disease (Supplementary Table S1).

All animals with detectable OROV antigen in the brain had widespread OROV immunohistochemistry signal in their spinal cords. The signal was associated with greater than 50% of neurons in all animals, and those were preponderantly motor neurons (Figure 1E and Supplementary Figure S1) no signals were detected in dorsal root ganglia. OROV antigen was not detected in the spinal cord alone, in absence of brain signal.

In order to check for the presence of OROV antigen in peripheral nerves, an extra group of mice was subcutaneously inoculated in the ventral thorax, above the rib cage. This is a procedure used to facilitate the search for viral antigen associated with easily identifiable intercostal nerves, which run parallel to the ribs. Although several sections were examined, no OROV antigen was detected in peripheral nerves (Figure 1F). *Bunyaviridae* replication and assembly occurs in association with the Golgi, therefore, if OROV replicates in peripheral neurons, structural viral antigens should be more abundant in cellular bodies. Thus, immunohistochemistry maybe be insensitive for the detection of low amounts of antigen moving along a few axons, and the presence of OROV in peripheral nerves will require further investigation.

Viruses that cause encephalitis have multiple strategies to reach the CNS, the two main routes being invasion through a neural route, such as rabies and herpes simplex viruses [15,16], and/or traversing the blood-brain barrier [17]. Viruses that adopt a neural route initially infect peripheral nerves, then the spinal cord, to be able to advance towards the brain. In this regard, Rift Valley fever virus, a *Bunyaviridae* in the genus *Phlebovirus*, leaves the undamaged the BBB of experimentally infected mice [18], while hantavirus may sporadically cause encephalitis with obvious capillary leakage in humans [19,20,21]. The present study suggests that OROV uses the neural route during early phases, reaching first the spinal cord, and later the brainstem and the remaining of the brain around 3 dpi. However, with the progression of infection, somehow OROV may later become able to cross the blood-brain barrier, which may happen in parallel with the neural spread and may be associated with viremia. What the present study indicates is that there is leakage through the barrier in later phases, when the virus had already reached the brain, as previously demonstrated for other agents, such as the flavivirus tick-borne encephalitis virus [22]. This would explain the presence of OROV antigen in non-contiguous sites, by a pattern of infection that will include early OROV progression along the neuronal route, from the spinal cord and then the brainstem, combined in later phases with virus movement through the blood-brain barrier, coinciding with the more pronounced clinical features of disease in this model.

This study documents the invasion of the CNS by OROV, combining features of infection through neural route with breach of the blood-brain barrier. Future studies might provide additional details of the OROV infection, which may reveal patterns of progression at the level of specific nuclei and cortical layers. The development of fluorescently tagged OROV may allow such detailed investigations to reveal OROV infection’s circuitry, and perhaps to develop its potential application as a viral vector for specific neuronal delivery of gene products.

## Supplementary Files

Supplementary File 1
