# Supplementary material for: Spread of Oropouche Virus into the Central Nervous System in Mouse
Source: Viruses. 2014 Oct 10;6(10):3827–36. doi: 10.3390/v6103827 (PMC4213564; doi:10.3390/v6103827)
Supplement: Supplementary File 1 [file viruses-06-03827-s001.pdf]

Supplementary Material

Spread of Oropouche Virus into the Central Nervous System in Mouse

Rodrigo I. Santos, Lézio S. Bueno-Júnior, Rafael N. Ruggiero, Mariana F. Almeida, Maria L. Silva, Flávia E. Paula, Vani M. A. Correa and Eurico Arruda

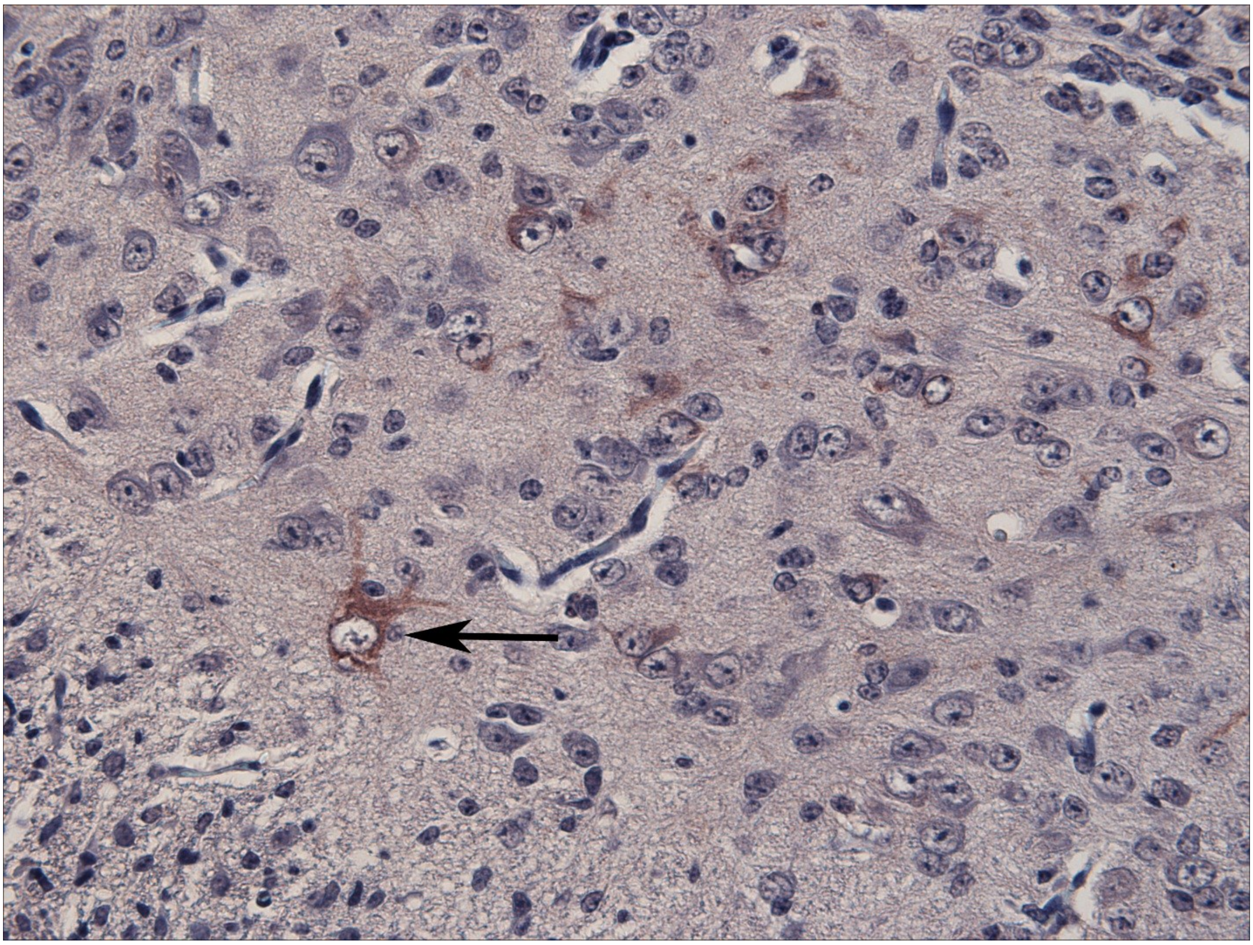

Supplementary Figure S1. OROV in mouse spinal cord. Magnification 400×. Arrows indicates infected motor neuron.

Supplementary Table S1. Retention of Evans Blue in the brain of infected and control animals.

ANIMAL	DO 560nm	symptoms
**Control 1**	0.094	none
**Control 2**	0.134	none
**Control 3**	0.106	none
**Control 4**	0.188	none
**Infected 1**	0.584	Paralysis, inability to feed, moribund
**Infected 2**	0.202	none
**Infected 3**	0.145	none
**Infected 4**	0.306	none
**Infected 5**	0.782	Paralysis, inability to feed, moribund
